# Supplementary material for: Reductive Transformation of O-, N-, S-Containing Aromatic Compounds under Hydrogen Transfer Conditions: Effect of the Process on the Ni-Based Catalyst
Source: Molecules. 2023 Oct 12;28(20):7041. doi: 10.3390/molecules28207041 (PMC10609389; doi:10.3390/molecules28207041)
Supplement: Supplementary file 1 [file molecules-28-07041-s001.zip › molecules-2635980-supplementary.pdf]

Reductive Transformation of O-, N-, S-Containing Aromatic Compounds under Hydrogen Transfer Conditions: Effect of the Process on the Ni-Based Catalyst

Nikolai S. Nesterov, Vera P. Pakharukova, Alexey A. Philippov, Igor P. Prosvirin *, Anton S. Shalygin and Oleg N. Martyanov

Boreskov Institute of Catalysis SB RAS, Academician Lavrentiev Ave. 5, Novosibirsk 630090, Russia;
science@catalysis.ru (N.S.N.); verapakharukova@yandex.ru (V.P.P.); philippov@catalysis.ru (A.A.P.);
antonchem86@mail.ru (A.S.S.); oleg@catalysis.ru (O.N.M.)
* Correspondence: prosvirin@catalysis.ru

Supplementary Materials

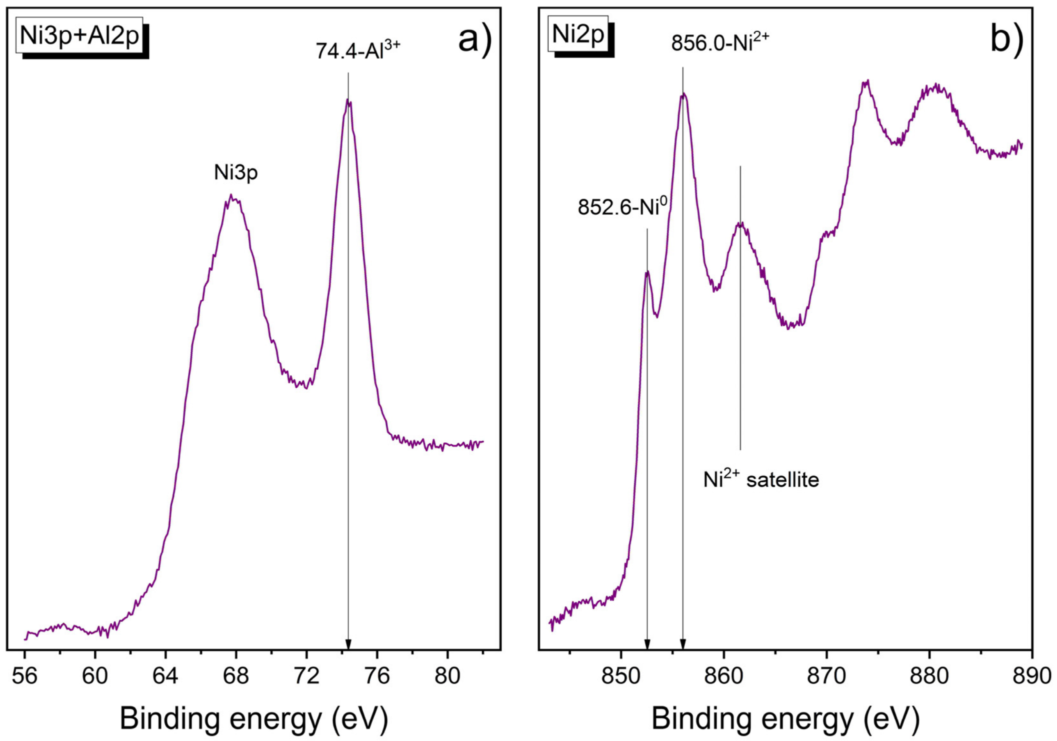

Figure S1. Ni3p + Al2p (a) and Ni2p (b) XPS spectra of catalyst after naphthalene conversion.

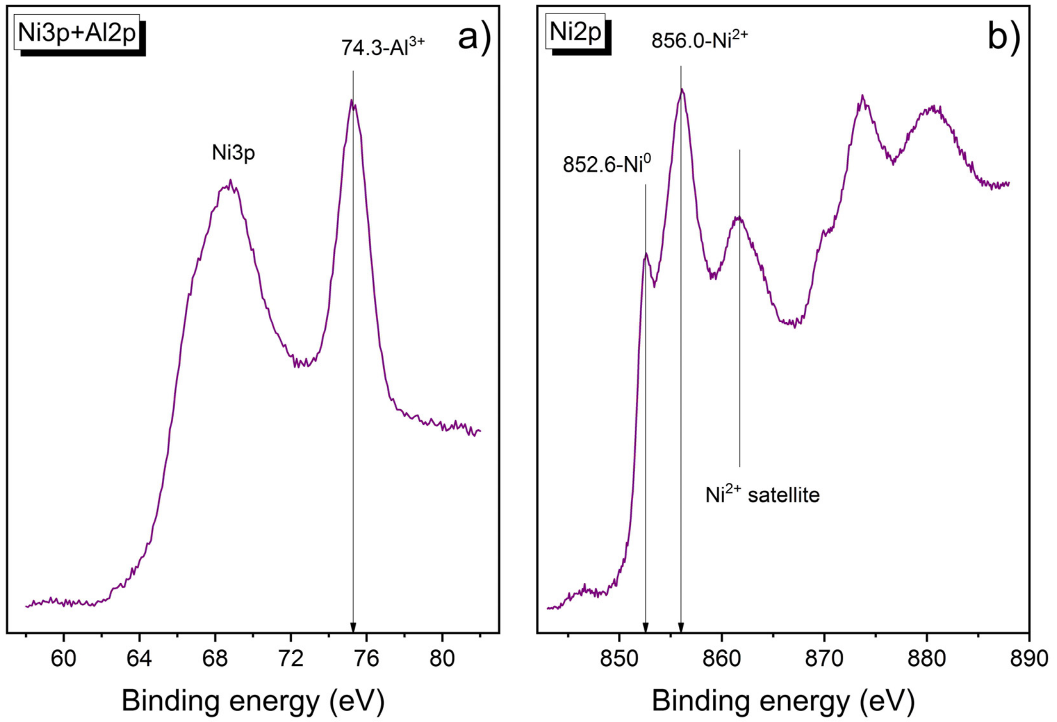

Figure S2. Ni3p + Al2p (a) and Ni2p XPS spectra of catalyst after 1-benzofuran conversion.

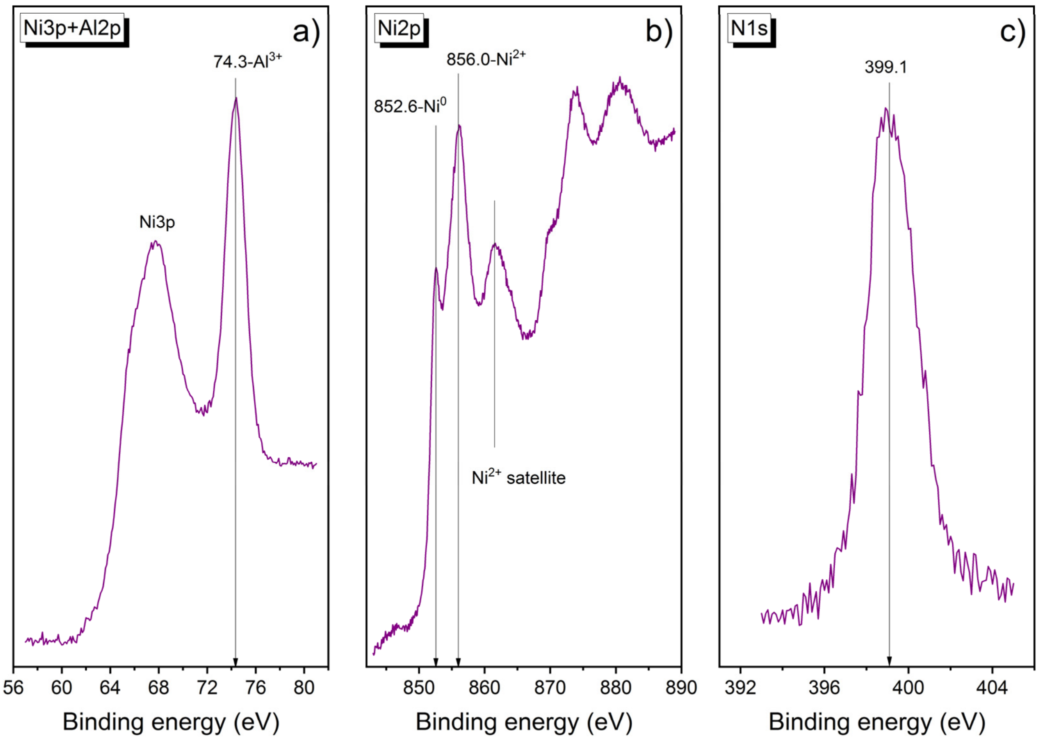

Figure S3. Ni3p + Al2p (a), Ni2p (b) and N1s (c) XPS spectra catalyst after indole conversion.

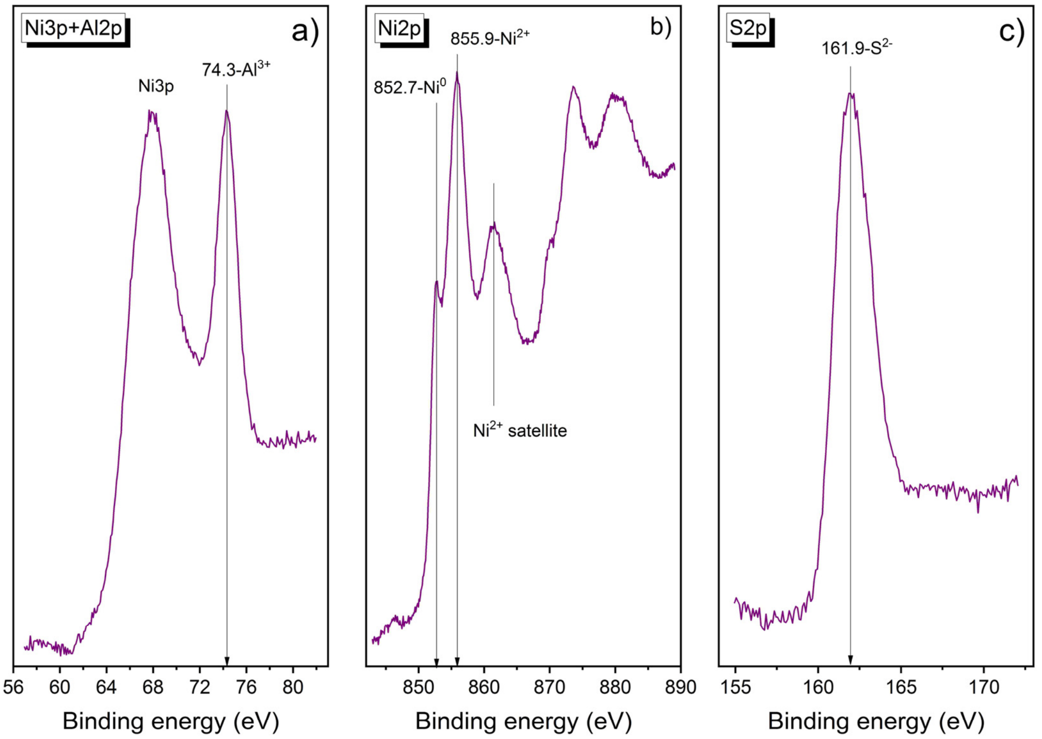

Figure S4. Ni3p + Al2p (a), Ni2p (b) and S2p (c) XPS spectra of catalyst after 1-benzothiophene conversion.

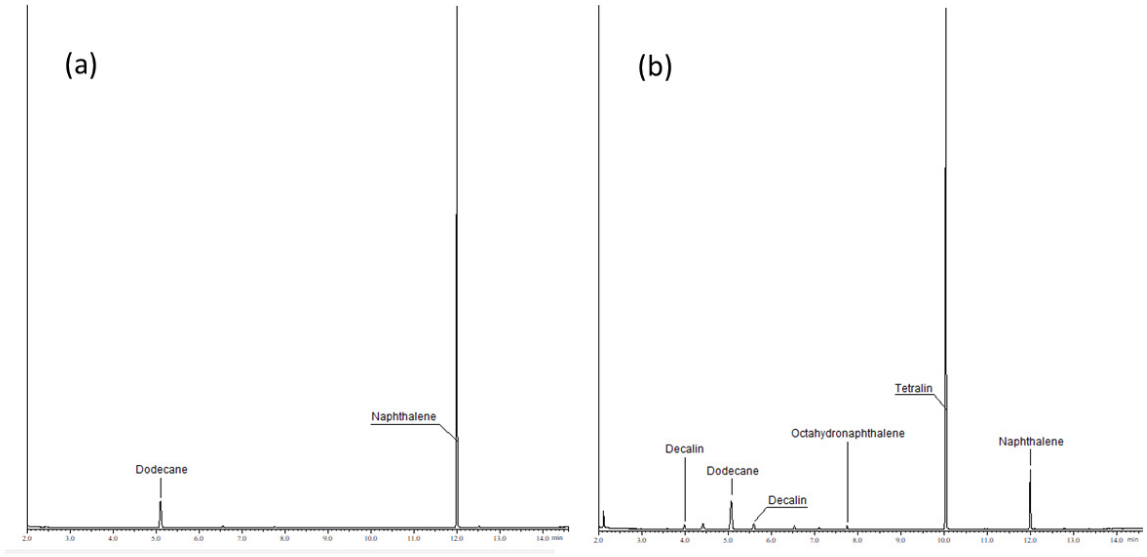

Figure S5. Chromatograms of the initial (a) and final (b) samples during the conversion of naphthalene.

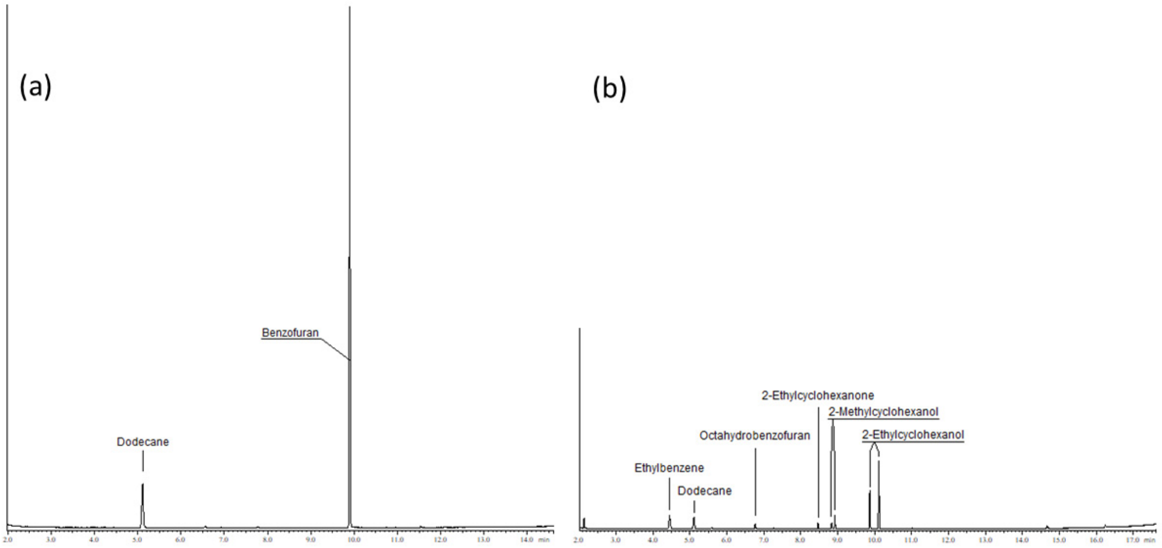

Figure S6. Chromatograms of the initial (a) and final (b) samples during the conversion of 1-benzofuran.

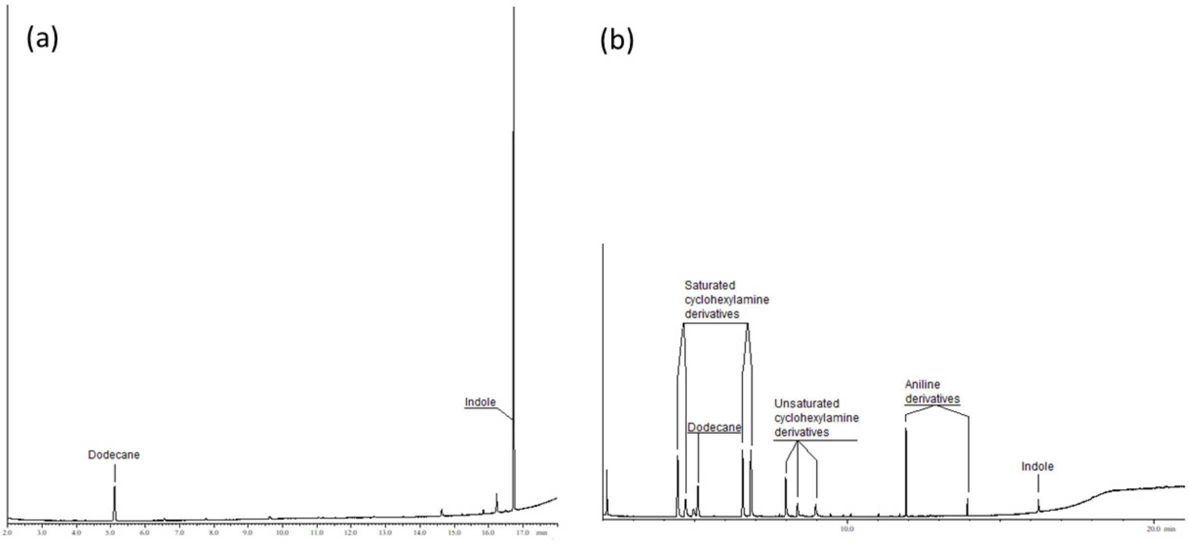

Figure S7. Chromatograms of the initial (a) and final (b) samples during the conversion of indole.

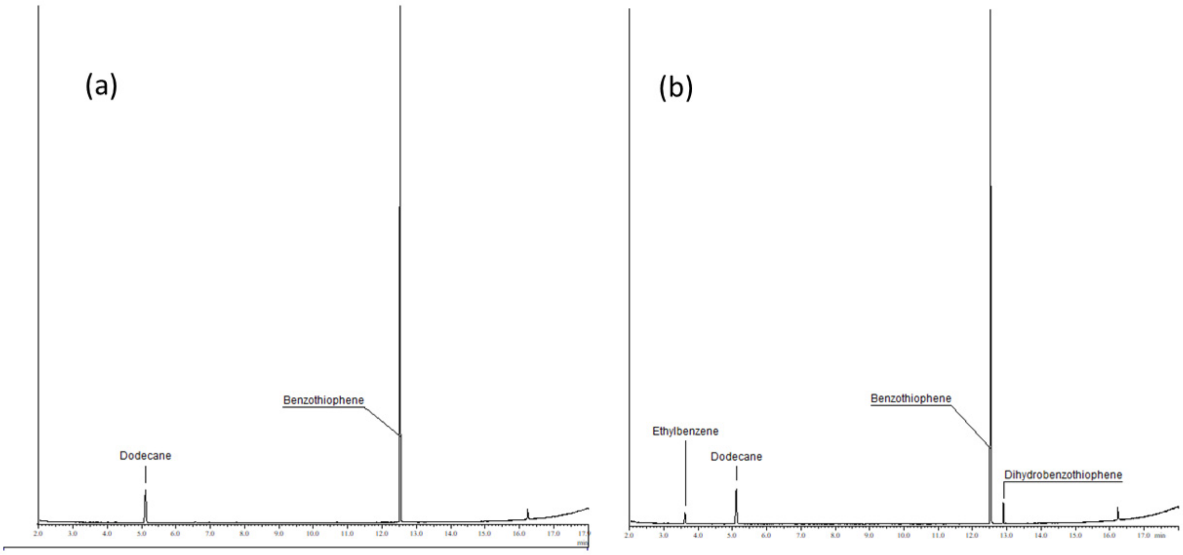

Figure S8. Chromatograms of the initial (a) and final (b) samples during the conversion of 1-benzothiophene.
